# The association between fasting plasma glucose and glycated hemoglobin in the prediabetes range and future development of hypertension

**DOI:** 10.1186/s12933-019-0859-4

**Published:** 2019-04-27

**Authors:** Mika Geva, Gadi Shlomai, Anat Berkovich, Elad Maor, Avshalom Leibowitz, Alexander Tenenbaum, Ehud Grossman

**Affiliations:** 1 Department of Internal Medicine D, Chaim Sheba Medical Center, Ramat Gan, Israel; 2 Sackler School of Medicine, Tel-Aviv University, Tel Aviv-Yafo, Israel; 3 Leviev Heart Center, Chaim Sheba Medical Center, Ramat Gan, Israel

**Keywords:** Prediabetes, HbA1c, Impaired fasting glucose, Hypertension, Insulin resistance, Cardiovascular risk

## Abstract

**Background:**

Prediabetes is a well-established risk factor for progression to overt diabetes mellitus (DM), which is in turn associated with development of hypertension (HTN) and vice versa. However, the role of prediabetes and HbA1c in particular as an independent risk factor for the development of hypertension is unclear.

**Aim:**

In this current study, we aimed to evaluate the association between both fasting plasma glucose (FPG) and hemoglobin A1c (HbA1c) levels in the prediabetes range and development of HTN among a large cohort of normotensive subjects.

**Design and methods:**

We investigated 5016 normotensive participants without DM and other cardiovascular risk factors who were annually screened in a tertiary medical center. Subjects were divided into normoglycemic and prediabetic groups. Normoglycemia was defined as HbA1c < 5.7% and FPG < 100 mg/dl. Prediabetes was defined according to the ADA criteria, i.e., 6.5% > HbA1c ≥ 5.7% or impaired fasting glucose (IFG):126 mg/dl > FPG ≥ 100 mg/dl. Subgroup analysis was made by dividing participants into four groups according to FPG and HbA1C levels, i.e., normoglycemia, impaired HbA1c only, IFG only, and both parameters impaired.

**Results:**

During a follow-up of 3.7 ± 2.9 years, 318 (6.3%) subjects developed HTN. A cumulative hazard function for the development of hypertension showed a 2.89-fold ([95% CI 2.19–3.83], p < .0001) increased risk for HTN in the prediabetic population. In a multivariable Cox proportional hazard regression model adjusted to common confounding risk factors for HTN, prediabetes was found to be independently associated with a 1.95-fold ([95%, CI 1.43–2.52] p < .0001) increased risk for hypertension. Impaired HbA1C only was not found to be independently associated with HTN, while IFG only showed a 2.13-fold (95%, [CI 1.46–3.11] p < .0001) increased risk for HTN compared to normoglycemic, and a 2.55-fold ([95% CI 1.85–3.51] p < .0001) increased risk for HTN when both parameters impaired.

**Conclusion:**

Our study demonstrates that FPG in the prediabetes range, albeit not glycated hemoglobin, is independently and significantly associated with future development of HTN. Therefore, our findings further highlight the pivotal predictive role of IFG for HTN development as opposed to the limited independent role of abnormal HbA1c levels.

**Electronic supplementary material:**

The online version of this article (10.1186/s12933-019-0859-4) contains supplementary material, which is available to authorized users.

## Introduction

Hypertension (HTN) and diabetes mellitus (DM) are very common pathological entities, which very frequently co-exist [1–3] Both DM and HTN are associated with increased cardiovascular (CV) morbidity and mortality [4]. HTN and DM demonstrate substantial similarities in their clinical manifestations as well as the underlying pathophysiological mechanisms, such as inflammation and oxidative stress, obesity, and insulin resistance [1, 5, 6]. As much as one-third of hypertensive subjects are also insulin resistant and fulfill the diagnostic criteria for the metabolic syndrome [7]. The American Diabetes Association (ADA) defines prediabetes as either impaired fasting glucose (IFG) (i.e., fasting glucose levels of 100–125 mg/dl), impaired glucose tolerance (IGT) (i.e., glucose levels post 2-h oral glucose loading of 140–199 mg/dl), or an elevated hemoglobin A1c (HbA1c) level of 5.7% to 6.4% [8]. Prediabetes is a well-established risk factor for progression to overt DM [8], which is in turn associated with development of HTN [1, 9] and vice versa [10]. However, the role of prediabetes as an independent risk factor for the development of HTN necessitates further elucidation [11–19]. Furthermore, data are scarce and somewhat conflicting regarding the specific prognosticator role of HbA1c in the prediabetic range and the development of future HTN [17–20]. Therefore, in this current study, we aimed to evaluate the association between both fasting glucose and HbA1c levels in the prediabetes range and development of HTN among a large cohort of normotensive subjects.

## Methods

### Study population

The Chaim Sheba Medical Center Institute for Medical Screening performs approximately 10,000 annual examinations. The data source for this study is a computerized database established in 2000, to which all data are recorded. All participants are asymptomatic men and women examined annually at the Chaim Sheba Medical Center Institute for Medical Screening. All participants were outpatients referred by their insurance company and/or their employer. The annual examination includes filling a standard questionnaire regarding demographic characteristics, a complete medical history, lifestyle and health-related habits, and any unusual medical events which occurred since the previous encounter. The height and weight of all participants, wearing light clothing without shoes, were measured and recorded at each visit. Subjects underwent a thorough physical examination, and blood pressure (BP) was measured in the seated position after a 3 min rest period using a standard sphygmomanometer. The body mass index (BMI) was calculated as weight in Kg divided by the height in m^2^.

All participants had extensive Blood tests performed, such as complete blood count and a comprehensive metabolic panel, including but not limited to renal and liver function testing, serum electrolytes, FPG, HbA1c levels and additional analyses as needed.

The study was approved by the Chaim Sheba Medical Center ethical Helsinki board according (Approval no. 4451-17-SMC). Data were recorded anonymously. No individual consent was obtained.

### Inclusion and exclusion criteria

The complete database included 11,630 individuals with measurements of fasting plasma glucose (FPG) and HbA1c. Individuals were excluded if they had HTN (n = 3624) or DM (n = 1266) at baseline or had only one clinic visit (n = 3025). HTN diagnosis was made based on prior diagnosis or use of anti-hypertensive medications. Participants without HTN but with BP readings ≥ 140 mmHg for systolic blood pressure (SBP) and/or ≥ 90 for diastolic blood pressure (DBP) (n = 744) were included.

**Fig. 1:**
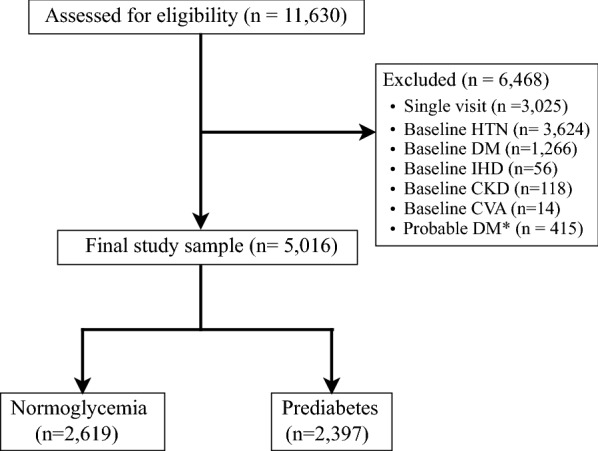
Flowchart of study participants. * No known DM, fasting plasma glucose > 126 mg/dl or HbA1c ≥ 6.5%. *HTN* hypertension, *DM* diabetes mellitus, *IHD* ischemic heart disease, *CKD* chronic kidney disease, *CVA* cerebrovascular accident

Participants without a formal diagnosis of DM but with glycemic indices suggestive of DM (i.e., FPG ≥ 126 mg/dl or HbA1c ≥ 6.5%) were excluded as well (n = 415). We also excluded patients with co-morbidities associated with HTN such as ischemic heart disease (IHD, n = 56), chronic kidney disease (CKD, n = 118) and cerebrovascular events (CVA, n = 14). Thus, the final study cohort comprised 5016 normotensive participants without DM (Fig. 1).

### Definitions and outcome measures

The primary outcome was the new onset of HTN. Subjects were considered to develop hypertension when, according to their primary care physicians, they had a new diagnosis of hypertension or started using antihypertensive medications. Patients with two separate BP readings ≥ 140 mmHg for SBP and/or ≥ 90 for DBP were referred for further evaluation by their primary care physician. Normoglycemia was defined as HbA1c < 5.7% and FPG < 100 mg/dl. Prediabetes was defined according to the ADA criteria, i.e., HbA1c ≥ 5.7% and < 6.5% or FPG ≥ 100 mg/dl and less than 126 mg/dl. DM was defined as either fasting plasma glucose greater than or equal to 126 mg/dl on two separate readings, self-reported history of DM or when the subject used insulin or oral hypoglycemic medications. Hypercholesterolemia was defined as total cholesterol of > 250 mg/dl, or a self-reported history of cholesterol-lowering medications use. Self-reported smoking status and physical activity level of participants were also obtained.

### Laboratory analysis

All biochemical analyses were performed on fresh samples in the Chaim Sheba Medical Center, Tel Hashomer core laboratory facility, using the Olympus AU2700TM Chemistry-Immuno Analyzer (Olympus; Shinjuku, Tokyo, Japan).

### Statistical analysis

Continuous data were compared with the Student t-test and 1-way analysis of variance. Categorical data were compared with the use of the χ^2^ test or Fisher exact test. We conducted Cox proportional hazard analysis to estimate the hazard ratio (HR) and 95% Confidence Interval (CI)s for developing HTN. We adjusted all groups for age, gender, statin use, smoking, physical activity, BMI, low-density lipoprotein (LDL) cholesterol, high-density lipoprotein (HDL) cholesterol, triglycerides (TG), and systolic and diastolic BP. Further validation of confounders was conducted by multivariable interaction analysis. Statistical significance was accepted for a 2-sided p < .05. The statistical analyses were performed with IBM SPSS version 20.0 (Chicago, IL).

## Results

The study population comprised 5016 individuals of whom 69.4% were men (Table 1). Mean follow up was 3.7 ± 2.9 years and mean age was 53.5 ± 9.9 years (Table 1). Among study participants 2619 (52.2%) were normoglycemic, and 2397 (47.8%) had prediabetes (Table 1). Notably, normoglycemic participants were significantly younger, were more likely to be women, had lower BMI, as well as lower SBP and DBP, lower TG levels and higher HDL levels compared with participants with prediabetes (Table 1). Those with prediabetes also had higher rates of statin use and lower rates of physical activity. There were no clinically significant differences between study groups concerning LDL levels and smoking status (Table 1).

**Table 1:** Baseline characteristics of study population

	Total n = 5016	Normal n = 2619	Prediabetes n = 2397	p value
Age^a^ (year)	53.5 (9.9)	51.03 (9.7)	55.94 (9.7)	p < .0001
Male gender^b^ (%)	3476 (69.4)	1678 (64.1)	1798 (75.2)	p < .0001
FPG (mg/dl)^a^	94.00 (10.72)	88.24 (7.49)	100.12 (10.27)	p < .0001
HbA1C %	5.5	5.2	5.8	p < .0001
BMI^a^	25.90 (3.75)	25.26 (3.73)	26.61 (3.64)	p < .0001
SBP (mmHg)^a^	120 (15)	117 (14)	122 (16)	p < .0001
DBP (mmHg)^a^	74 (10)	72 (10)	76 (10)	p < .0001
Statin use^b^ (%)	181 (3.6)	75 (2.9)	106 (4.4)	p = .004
Current smoker^b^ (%)	729 (14.4)	387 (14.8)	342 (14.4)	p = .688
Physically active^b^ (%)	3734 (74.7)	1995 (76.3)	1739 (73)	p = .008
LDL (mmole/l)^a^	122 (28)	122 (27)	122 (28)	p = .869
HDL (mmole/l)^a^	50 (13)	52 (14)	48 (12)	p < .0001
Triglycerides (mmole/l)^a^	121 (64)	113 (64)	129 (63)	p < .0001

*FPG* fasting plasma glucose, *BMI* body mass index, *SBP* systolic blood pressure, *DBP* diastolic blood pressure, *LDL* low-density lipoprotein, *HDL* high-density lipoprotein

^a^Values are expressed as mean ± SD

^b^Values are expressed as absolute number (percentage of group)

### Prediabetes is an independent risk factor for HTN

**Fig. 2:**
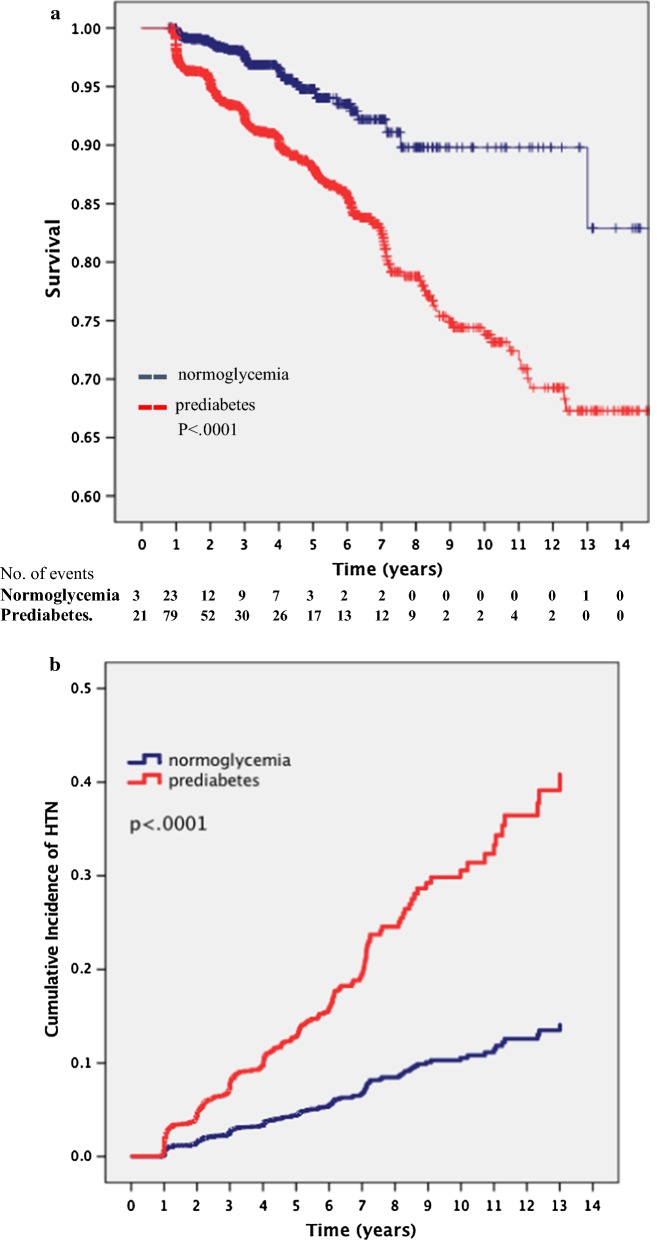
**a** Kaplan–Meier curve showing the probability of HTN event-free survival. Number of events (new onset HTN) are documented at the bottom of the table, according to normoglycemic and prediabetic groups. p value (log rank) < .0001. **b** Cumulative incidence of hypertension during follow up according to normoglycemia and prediabetes states, p-value (log-rank) < .0001

HTN developed in 318 participants (6.3%), 62 (2.4%) in the normoglycemia group and 269 (11.2%) in the prediabetes group. Kaplan–Meier survival analysis (Fig. 2a) showed that the cumulative probability for the development of HTN at 6 years of follow up was 8% in the normoglycemic group and 17% in the prediabetic group, reaching up to 10% and 33% at 12 years of follow up respectively, log-rank test p < .0001 for the overall difference between two groups. A cumulative hazard function for the development of hypertension showed a 2.89-fold ([95% CI 2.19–3.83], p < .0001) increased risk for HTN in the prediabetic population (Fig. 2b).

In a multivariable Cox proportional hazard regression model adjusted to common confounding risk factors for HTN, prediabetes was found to be independently associated with a 1.95-fold 95%, CI 1.43–2.52] p < .0001) increased risk for HTN (Table 2).

**Table 2:** Multivariable Cox regression model for the outcome of hypertension

	HR	95% CI	p
Prediabetes vs. normoglycemic	1.95	1.46–2.62	*<* .0001
Age (each year)	1.01	.99–1.025	.066
Male sex	1.15	.83–1.59	.388
BMI	1.06	1.02–1.09	< .0001
SBP	1.03	1.02–1.04	< .0001
DBP	1.03	1.02–1.05	< .0001
LDL	.59	.99–1.00	.996
HDL	.21	.98–1.00	.993
Triglycerides	.73	.99–1.00	1.00
Statin use	1.27	.72–2.07	.346
Physically active	1.18	.91–1.53	.204
Smoking	1.10	.78–1.55	.569

*FPG* fasting plasma glucose, *BMI* body mass index, *SBP* systolic blood pressure, *DBP* diastolic blood pressure, *LDL* low-density lipoprotein, *HDL* high-density lipoprotein

Additional independent associated risk factors for hypertension were systolic, and diastolic blood pressure, with every increment of 1 mmHg, was associated with a corresponding of 3.2% and 4% increased risk for HTN respectively, so as BMI with an increment of 1 unit of BMI was found to have a 5% increased risk for HTN. Other covariates such as age, gender, lipid profile, and statin use did not show any significance (Table 2). Interaction analysis for confounders factors: age, sex, BMI, LDL, HDL triglycerides, statin use, smoking and physically active demonstrated that the association of prediabetes with HTN was consistent in all subgroup analyzed suggesting an independent association.

### The association of different measures of glycemic control and their association with HTN

We divided the study population into four groups according to their FPG and HbA1c levels. Group 1 included participants with normal levels of both FPG and HbA1C (FPG < 100 mg/dl HbA1c < 5.7%) (n = 2619). Group 2 included participants with normal FPG but impaired HbA1C (FPG < 100 mg/dl and 5.7% ≤ HbA1c < 6.5%) (n = 974). Group 3 included participants with normal HbA1c values but with IFG (100 ≤ FPG < 126 mg/dl and HbA1c < 5.7%) (n = 595) and group 4 included subjects with both parameters impaired in the prediabetes range (100 mg/dl ≤ FPG < 126 mg/dl and 5.7% ≤ HbA1c < 6.5%) (n = 828). Baseline characteristics for the subjects in the different sub-analysis groups are shown in Table 3.

**Table 3:** Baseline characteristics of participants according to FPG and HbA1c levels

	Group 1 normal FPG, normal HbA1c (n = 2619)	Group 2 normal FPG, impaired HbA1c (n = 974)	Group 3 impaired FPG, normal HbA1c (n = 595)	Group 4 impaired FPG, impaired HbA1c (n = 828)
Age (year)^a,‡^	51.03 (9.73)	56.79 (9.84)	53.88 (9.15)	56.42 (8.79)
Male gender (%)^b,‡^	1678 (64.1)	653 (67.3)	482 (81.1)	663 (80.3)
FPG (mg/dl)^a^	88.24 (7.49)	91.0 (8.31)	105.15 (5.05)	107.24 (6.25)
HbA1C %^a^	5.2 (3.7)	5.9 (3.7)	5.3 (3.5)	6.0 (3.6)
BMI^‡^	25.26	26.15	26.50	27.21
SBP (mmHg)^a,‡^	117 (14)	121 (15)	123 (17)	124 (16)
DBP (mmHg)^a^	72 (10)	75 (10)	76 (11)	77 (10)
Statin use (%)^b^	75 (2.9)	46 (4.7)	18 (3.0)	42 (5.1)
Current smoker (%)^b^	387 (14.8)	163 (16.8)	58 (9.8)	121 (14.7)
Physically active (%)^b^	1995 (76.3)	705 (72.8)	425 (71.9)	609 (74.2)
LDL (mmole/l)^a^	122 (27)	122 (28)	123 (26)	121 (29)
HDL (mmole/l)^a,‡^	52 (14)	50 (13)	47 (11)	47 (11)
Triglycerides (mmole/l)^a, ‡^	113 (64)	123 (59)	129 (66)	137 (64)

*FPG* fasting plasma glucose, *BMI* body mass index, *SBP* systolic blood pressure, *DBP* diastolic blood pressure, *LDL* low-density lipoprotein, *HDL* high-density lipoprotein

^a^Values are expressed as mean ± SD

^b^Values are expressed as absolute number (percentage of group)

^‡^p-value for trend < .05 for comparison between group 2 and group 3

In a multivariable Cox proportional hazard regression model subjects in group 2 did not show a significant hazard risk for HTN compared to group 1 (Table 4), while group 3 was associated with a 2.13-fold (95%, [CI 1.46–3.11] p < .0001) increased risk for HTN compared to group 1 (Table 4). Participation in group 4 was associated with a 2.55-fold ([95% CI 1.85–3.51] p < .0001) increased risk for HTN compared to group 1 (Table 4). When assessed as a continuous covariate in a multivariable Cox proportional hazard regression model each 1 mg/dl increment in FPG was associated with a corresponding 4.6% increased risk for HTN (HR 1.04, CI 95%1.03–1.06, p < .0001), whereas an increment in HbA1c level was not significantly associated with an increased risk for HTN.

**Table 4:** Multivariable Cox regression model for the outcome of hypertension according to groups

	HR	95% CI	p
Group 2 vs. Group1	1.27	.87–1.81	.213
Group 3 vs. Group 1	2.13	1.46–3.11	< .0001
Group 4 vs. Group 1	2.55	1.85–3.51	< .0001

Multivariate model was adjusted for: gender, age, BMI, SBP, DBP, LDL, HDL, TG, physical activity smoking and statin use

*BMI* body mass index, *SBP* systolic blood pressure, *DBP* diastolic blood pressure, *LDL* low-density lipoprotein, *HDL* high-density lipoprotein, *TG* triglycerides

Finally, we conducted subgroup analysis, with a stricter exclusion of possible undiagnosed baseline HTN in which in addition to the exclusion criteria of HTN diagnosis or hypertensive medication we also excluded participants who only had BP readings ≥ 140 mmHg for SBP and/or ≥ 90 mmHg for DBP in their first visit (n = 744) (Additional file 1: Table S1). The same analysis was conducted in this subgroup population (n = 4272). The results were the same to all analysis with even a higher hazard ratio for the development of HTN in the prediabetic group compare to the normoglycemic group (Additional file 1: Table S1), as well as in the four groups analysis (Additional file 1: Table S2).

## Discussion

In this large retrospective cohort study, we demonstrated an independent significant association between FPG levels in the prediabetes range and future development of HTN. These findings further emphasize the role of insulin resistance in the pathogenesis of HTN. Several plausible pathophysiological mechanisms may underlie the association of impaired glycemic control and development of HTN; Elevated FPG has been previously associated with arterial stiffness [21–23], most likely via oxidative stress and accumulation of glycation end products, and alterations in activities of vasoactive substances [24–26]. Furthermore, in patients with DM, the integrity of the vascular wall is more susceptible to damage, specifically in the presence of CV risk factor and these macrovascular changes are also evident in the prediabetic phase [27, 28]. In addition to the well-established effects of insulin resistance and hyperglycemia on the macrocirculatory dysfunction, one must not ignore the role of glycemic dysregulation on the microcirculation. In particular, its role in the development of endothelial dysfunction and alterations in vascular extracellular matrix most likely secondary to inflammatory and pro-fibrotic alterations [28]. IFG and glycated hemoglobin represent different pathophysiological aspects of glycemic control; whereas IFG expresses increased hepatic glucose production in the fasting state, HbA1c encompasses glycemic control in the fasting state as well as post-prandial glucose levels [29]. Therefore, we also assessed the differential risk for HTN development among patients with prediabetes diagnosed by either isolated HbA1C criterion, isolated IFG criterion or both criteria. We found that elevated IFG was independently associated with increased risk of HTN, whereas impaired HbA1c as a sole criterion for prediabetes (i.e., with normal FPG) was not independently associated with increased risk for HTN. While HbA1c is a very useful tool in the diabetes management armamentarium, it has few notable caveats. First, it is a fairly crude measurement of glycemic control, and as such a single HbA1c percentage may represent a wide spectrum of glucose levels [8]. Second, because HbA1c represents the average glycated hemoglobin level, it often fails to properly account for extreme glucose values and for the glucose variability as well [8]. Furthermore, HbA1C levels are susceptible to false elevations and false reductions secondary to various medical comorbidities and substance abuse [8]. Several studies have previously described the association between IFG and HTN development [11–19]. However, these studies have mainly been conducted among Asian participants [12, 14–17, 19]. While Lee et al. found no association between IFG, IGT and HTN [12], other studies in Asian subjects found IFG to be an independent risk factor for development of HTN [14–17, 19]. There are also studies conducted among Caucasians participants; however, these studies yielded conflicting results [11, 13, 30]. Interestingly, a predictive association of elevated fasting serum insulin with incident HTN in European Americans has also been previously reported [31]. As previously mentioned, FPG and HbA1c levels identify different pathological abnormalities in glucose metabolism [32–34]. We currently found that individuals who had isolated impaired HbA1c had a distinctly different profile. They were significantly older and more likely to be women, had lower BMI, higher HDL cholesterol levels but lower TG levels and lower SBP compared with those who had IFG with normal HbA1c (Table 3). Similar findings were also previously reported in a large study of patients without DM [35]. The prognosticator role of HbA1c in the incidence of cardiovascular morbidity and mortality events is unclear; its’ associated risk is mostly attributed to confounding risk factors [36–38]. In a recent Japanese population study, Kuwabara et al. did not find HbA1c to be an independent risk factor for developing HTN, whereas each 10 mg/dl increase in FPG was associated with a 42.2% increased risk for developing HTN over 5 years [17]. A similar pattern of a differential association of IFG rather than elevated HbA1c and incident HTN was also previously reported by Heianza et al. [19]. In addition, Britton et al. have shown that in women without diabetes HbA1c levels were not significantly associated with increased risk of HTN after adjustment for BMI [20]. These findings concur with the results of our current study showing a 4.6% increased risk for HTN for every 1 mg/dl increment of FPG and a twofold increased risk for HTN independently associated with IFG. Notably, in our study, the risk for HTN increased significantly by 19% when both measures of glycemic control, i.e., FPG and HbA1C were both impaired. Bower et al. have demonstrated that higher HbA1c in the prediabetic range was independently associated with incident self-reported HTN [18].

Our study is observational; hence we can only postulate regarding the underlying mechanisms responsible for the differential association of IFG and HbA1c and incident HTN. First, as HbA1c levels are influenced by the glycation of proteins in the body due to hyperglycemia, it has been suggested that glucose concentrations may more directly reflect the pathogenesis of the HTN development compared to HbA1c which is an indirect measure of dysglycemia [19]. Furthermore, a discordance between glucose level in the prediabetic or diabetic range and HbA1c values has been previously described by others [35, 39, 40], and thus may provide a possible explanation for our findings.

Our study has several strengths and limitations. First, our database relies on annual clinical and laboratory evaluation, which enables us to perform a time-based survival analysis for HTN development, which is expressed as Hazard Ratio, rather than a cumulative 5-years incidence rate as reported by previous studies [11–17, 19]. We hypothesize that our annual data gathering and analysis permits superior delineation of HTN development over time compared to a cumulative 5-years incidence rate. Unlike previous studies [11–17, 19], our definition of HTN was not based on a single BP measurement and in addition, we excluded the presence of white coat HTN and other etiologies that may cause falsely elevated BP measurement using a strict definition for new-onset HTN. To further emphasize and verify our results we performed a subgroup analysis with a stricter HTN free exclusion. Our findings support a higher hazard risk for HTN in our subgroup analysis as compared to the all-cohort analysis (Additional file 1: Tables S1, S2). There are several possible explanations for this finding. There could be a dominance of patients with white coat HTN, a paucity (n = 25) of extremely high BP levels, i.e., above 160/100 mmHg, or the relatively small number of participants and event rates in this group. Therefore, the subgroup analysis highlights our finding of prediabetes and IFG in particular as an independent and major risk factor for HTN. Our study has several limitations. First, this is a retrospective observational study. Second, our cohort comprises of middle-aged men and women with a higher-than-average socioeconomic status, higher rates of physically activity and lower BMI levels, all of which may limit the generalization capacity of our findings and attributes to the relatively low events rates of newly HTN in this cohort. In addition, our cohort had a relatively lower rate of women participants, which may preclude a significant sex effect on the development of HTN. Moreover, while our study is notable for using both PFG and HbA1C as indices of glycemic control, we did not have specific data regarding IGT.

## Conclusion

In conclusion, our study demonstrates that FPG in the prediabetes range, albeit not glycated hemoglobin, is independently and significantly associated with future development of HTN. Therefore, our findings further highlight the pivotal predictive role of IFG for HTN development as opposed to the limited independent role of abnormal HbA1c levels.

## Additional file

**Additional file 1.** Subgroup analysis: exclusion of possible undiagnosed baseline HTN. **Table S1.** Multivariable cox regression model for the outcome of hypertension in the subgroup analysis. **Table S2.** Multivariable cox regression model* for the outcome of hypertension in the subgroup analysis according to groups.

## Abbreviations

DM: diabetes mellitus
HTN: hypertension
FPG: fasting plasma glucose
HbA1c: hemoglobin A1c
IFG: impaired fasting glucose
IHD: ischemic heart disease
CKD: chronic kidney disease
CVA: cerebrovascular accident
BMI: body mass index
SBP: systolic blood pressure
DBP: diastolic blood pressure
LDL: low-density lipoprotein
HDL: high-density lipoprotein
TG: triglycerides
