# Supplementary material for: The association between fasting plasma glucose and glycated hemoglobin in the prediabetes range and future development of hypertension
Source: Cardiovasc Diabetol. 2019 Apr 27;18:53. doi: 10.1186/s12933-019-0859-4 (PMC6486972; doi:10.1186/s12933-019-0859-4)
Supplement: Supplementary file 1 — Additional file 1. Subgroup analysis: exclusion of possible undiagnosed baseline HTN. Table S1. Multivariable cox regression model for the outcome of hypertension in the subgroup analysis. Table S2. Multivariable cox regression model* for the outcome of hypertension in the subgroup analysis according to groups. [file 12933_2019_859_MOESM1_ESM.docx]

**Subgroup analysis (n=4,272): excluding baseline BP readings ≥140 mmHg SBP and/or ≥90 mmHg DBP**

Table S1. Multivariable cox regression model for the outcome of hypertension in the subgroup analysis

|  | **HR** | **95% CI** | **P** |
| --- | --- | --- | --- |
| **Prediabetes vs. normoglycemic** | **2.21** | **1.43-3.42** | **<.0001** |
| **Age (each year)** | **1.03** | **1.01-1.04** | **.002** |
| Male sex | 1.08 | 0.68-1.69 | .736 |
| **BMI** | **1.09** | **1.05-1.14** | **<.0001** |
| SBP | 1.01 | 0.98-1.03 | .342 |
| DBP | 1.01 | 0.98-1.04 | .389 |
| LDL (each 1 mmole/L) | 0.99 | 0.99-1.00 | .280 |
| HDL (each 1 mmole/L) | 0.99 | 0.97-1.01 | .441 |
| Triglycerides (each 1 mmole/L) | 1.0 | 0.99-1.00 | .983 |
| Statin use | 1.25 | 0.65-2.40 | .496 |
| Physically active | 1.19 | 0.81-1.74 | .366 |
| Smoking | 1.14 | 0.71-1.82 | .584 |

Abbreviation: FPG, fasting plasma glucose; BMI, body mass index; SBP, systolic blood pressure;

DBP, diastolic blood pressure; LDL, low‐density lipoprotein; HDL, high-density lipoprotein

|  | HR | 95% CI | P |
| --- | --- | --- | --- |
| Group 2 vs. Group 1 | 1.43 | 0.84-2.42 | .181 |
| Group 3 vs. Group 1 | 2.24 | 1.25-4.01 | .007 |
| Group 4 vs. Group 1 | 3.01 | 1.88-4.82 | <.0001 |

Table S2. Multivariable cox regression model* for the outcome of hypertension in the subgroup analysis according to groups

*Multivariate model was adjusted for: sex, age, BMI, SBP, DBP, LDL, HDL, TG, physical activity smoking and statin use. Abbreviation: BMI, body mass index; SBP, systolic blood pressure; DBP, diastolic blood pressure; LDL, low‐density lipoprotein; HDL, high-density lipoprotein; TG, triglycerides;
